# Subsea Gate Valve—PDC Material and Sand Slurry Test

**DOI:** 10.3390/ma18245546

**Published:** 2025-12-10

**Authors:** Mehman Ahmadli, Tor Berge Gjersvik, Sigbjørn Sangesland

**Affiliations:** Department of Geosciences, Norwegian University of Science and Technology, 7031 Trondheim, Norway; tor.b.gjersvik@ntnu.no (T.B.G.); sigbjorn.sangesland@ntnu.no (S.S.)

**Keywords:** gate valve, PDC, petroleum, production, subsea

## Abstract

**Highlights:**

**What are the main findings?**

- PDC coating reduced erosion depth by 77.6% compared with WC.
- Valve lifetime extended by about 4.5 times under slurry flow.
- Erosion concentrated near gate–seat interface areas.

**What are the implications of the main findings?**

- PDC coating improves durability of subsea gate valves.
- Lower wear reduces maintenance and downtime costs.
- Supports material selection for erosion-critical valve designs.

**Abstract:**

Produced well flow is controlled through valves placed in the Christmas tree. Being mostly gate-type valves, they isolate the well from the surface when commanded or automatically in an emergency. The reliability of these valves is essential for subsea wells, as maintenance and replacement involve high cost, time, and HSE risks. Their design must withstand harsh conditions such as high temperature, pressure, solid particles, and corrosive environments. However, failures caused by leakage, cold welding, and the erosion of sealing elements are still common. These issues motivated the initial stage of this research, which experimentally showed that replacing the current tungsten carbide (WC) coating with polycrystalline diamond compact (PDC) material reduces friction and wear due to its high hardness and thermal stability. Based on these results, a 3D subsea gate valve model was developed and simulated in Ansys Fluent 2024 R2 under API slurry test conditions using the Oka erosion and Discrete Phase Models. A comparative analysis of WC and PDC coatings for a 5-inch gate valve exposed to a 2% sand slurry (250–400 μm) showed that PDC reduces the erosion depth by 77.6% and extends the valve lifetime by 4.5 times. The findings support the use of PDC for improved erosion resistance in subsea valve applications.

## 1. Introduction

### 1.1. Background

According to the International Energy Agency (IEA) (2024) [1], global energy demand is projected to grow at an annual rate of 0.7% from 2023 to 2030. Currently, around 80% of this demand is satisfied by fossil fuels, and this trend is expected to peak around 2030, despite the advancements in the renewable energy sector. To meet the rising demand, fossil fuel exploration and production are moving towards deep- and ultra-deep-water locations, high-pressure, high-temperature (HPHT) wells, and marginal fields [2,3]. This move brings new challenges for the entire operation, including the need for field-proven valve designs. Using subsea valves in their current form in non-traditional fields may lead to various problems such as galling, cold welding, and seal failures [4,5]. Flowing solid particles, such as produced sand, boost the failure process. The coating layer on the seal area is exposed to solid particles with abrasive shapes and high kinetic energy. Additionally, sand particles are trapped between the valve gate and seats, which results in faster degradation of the subsea valve [6].

### 1.2. Industrial Challenge

Despite their smaller size compared to other elements of subsea production systems, the valves, when they fail, result in production halts, leading to significant maintenance expenses and lost profits [7]. This can be considered one of the primary challenges with valves. Generally, subsea valves are designed with a lifetime of 20 to 25 years [8]. However, maintenance or replacement may be necessary in practice, depending on the operating conditions. Service times are predictable in traditional fields due to available data; however, there is a lack of data in non-traditional fields [9].

An all-electric control system is considered for future production fields, where the valve actuator is all-electric, using an electric motor and gears to operate the valve [10]. By replacing the hydraulic actuator with the electric one, the operator can have benefits such as real-time monitoring, improved safety, precise and faster control, and the removal of hydraulic lines [11]. However, it must be noted that electric actuators tend to have a longer actuating duration, which increases the exposure of valve internals to flowing fluids and solid particles. As a result, a higher erosion rate decreases the lifetime of the subsea valve, and the predictability of valve maintenance or replacement remains a concern.

### 1.3. Research Question

The previous sections cover the challenges related to subsea valve operations. To address these challenges, we proposed using PDC material instead of the currently used WC material as a coating material. The proposal is based on the expectation that PDC material lowers the frictional force and offers high durability and wear resistance at the same time. Therefore, simulations and laboratory experiments were carried out to show that PDC material offers reduced actuation power and increased wear resistance [12,13]. A prototype will be manufactured to increase the TRL and undergo various tests. The most challenging test among them is a slurry sand test, where the valve is subjected to sand slurry and must operate under the maximum allowable leakage limit. Before making the prototype, it is economically beneficial to investigate how PDC material is expected to behave regarding the erosion rate in the slurry test conditions. One of the methods is getting data from the open literature; however, as given in Section 1.5, there is a gap in the literature addressing our specific needs regarding subsea gate valves. Therefore, it is decided to employ a simulation software—in our case, Ansys Fluent 2024 R2—to answer the following questions: what is the expected erosion rate and distribution of PDC coating in the subsea valve during the slurry test, and how does this affect the expected lifetime of the subsea gate valve? For comparison and benchmarking purposes, a WC-coated subsea valve is also simulated.

### 1.4. Material Properties

The properties of WC material have been well documented as it has been industry standard for subsea and topside valves. The tungsten coating may include cobalt, chromium, or nickel to enhance its parameters depending on the operating condition. For valve applications, cemented WC-Co coating is commonly used, with Vicker’s hardness range between 1200 and 1600 HV and self-paired friction between 0.12 and 0.20, depending on the surface finish and contact condition [14,15]. Thermal expansion varies between (4.3 and 6.0) × 10^−6^ K^−1^ within 25–800 °C, and it has relatively high thermal conductivity of 84–110 W m^−1^ K^−1^ [16,17]. The most common deposition techniques for cemented WC coatings include High-Velocity Oxy-Fuel (HVOF) and plasma spraying, both of which produce dense microstructures with strong adhesion to metallic substrates [18,19]. Among these, HVOF-sprayed WC–Co coatings are preferred for subsea valves due to their high particle velocity during spraying, resulting in a lower porosity (<1.5%) and higher bond strength (>70 MPa) compared with plasma-sprayed counterparts. The coating performance is further influenced by spray parameters, substrate roughness, and post-treatment such as grinding or polishing, which affect the surface finish and friction behavior [7].

Polycrystalline Diamond Compact (PDC), in contrast, is a composite structure consisting of a polycrystalline diamond (PCD) layer bonded to a tungsten carbide substrate, typically produced through High-Pressure High-Temperature (HPHT) sintering at about 5–6 GPa and 1400–1600 °C [20,21]. During the HPHT process, fine diamond grains form strong inter-crystalline bonds, while cobalt diffuses from the carbide substrate to catalyze diamond–diamond bonding. This results in a layer with exceptional hardness (7000–9000 HV), a low friction coefficient (0.05–0.08), and high thermal conductivity (600–1200 W m^−1^ K^−1^) [22,23].

A key challenge for PDC materials is the thermal expansion mismatch between diamond and the tungsten–carbide substrate. Upon cooling after HPHT sintering, this mismatch induces residual tensile stress at the interface, which can lead to delamination or microcracking if not properly managed [24,25]. To mitigate these effects, graded interlayers or binder-phase control are commonly employed to balance thermal stresses. The thermal stability of PDC strongly depends on the diamond grain size, cobalt content, and substrate thickness. Studies report that cobalt-catalyzed PDC begins to degrade above 700 °C, while silicon-bonded PDC variants maintain stability up to 1200 °C [22]. These characteristics make PDC coatings especially promising for high-load, erosive environments where conventional WC coatings are prone to wear and delamination. The comparative properties of WC and PDC coatings, together with applicable testing standards, are summarized in Appendix A (Table A1).

Prior microscopic observations by the authors under sliding wear conditions [13] showed distinct damage patterns between the coatings. WC exhibited abrasive grooves, microcracks along carbide–binder interfaces, and local grain pull-out, whereas PDC surfaces remained largely polished with no visible cracking or delamination. These behaviors are consistent with the intrinsic properties summarized above.

### 1.5. Related Work

There has been various research on developing models to relate the contact material and flow conditions to erosion. One of the earliest is Finnie’s model, which performs well with ductile materials but has deviations with brittle materials and complex geometries [26]. Meanwhile, McLaury extended Finnie’s model by incorporating fluid properties, which made it more suitable for pipeline applications [27]. However, in both models, particle impact angles are not addressed properly [6]. To address this limitation, Oka carried out extensive experiments and developed a model that is applicable to both ductile and brittle materials [28]. However, for more complex geometries and flow conditions, empirical formulas remain insufficient and less accurate compared to integrated computational fluid dynamic (CFD) analysis models.

In the literature, there are an uncountable number of research studies that adopted CFD in their investigation. Regarding the gate valves, Jong Hyok Ri modeled gate valve erosion under lubricated and non-lubricated conditions, revealing that lubrication significantly reduces erosion rates, particularly in small flow areas [6]. Subsea gate valves operate without any installed lubrication mechanism and instead rely on the produced oil fluid. For the gas-producing well, the valve contact is dry. However, the methods that are used bear their significance for our study. In another study, Ri proposed an analytical model to evaluate wear and structural stability during valve actuation, emphasizing the critical role of internal coatings [29]. Similarly, Messa validated CFD results with experiments to estimate the valve lifespan and introduced a formula for the integral erosion ratio based on flow velocity and valve opening [30]. There are many other studies on related topics, but they typically cover specific cases. We investigate the PDC material in the subsea gate valves and benchmark the results with WC material, which is missing in the current literature. Building on these studies, the present work applies CFD-based erosion modeling to a subsea gate valve using realistic slurry conditions defined by API 6A and extends the analysis to evaluate long-term wear progression through cumulative exposure assessment.

## 2. Methodology

### 2.1. Flow Model

The flow model of the simulation is based on the Reynolds-Averaged Navier–Stokes (RANS) equations. The RANS model consumes low computing power while being able to capture the turbulence effects through a turbulence model covered in Section 2.2 [31]. Comparatively, Unsteady Reynolds-Averaged Navier–Stokes equations offer benefits such as moving parts, oscillating flows, and varying loads; however, to optimize the running time and power, the RANS equations are preferred. Equations (1)–(4) describe the basis of steady-state turbulent flow—the conversation of mass and momentum, respectively. The equations are derived from the general equation for the simulation by assuming incompressible fluid, single-phase flow, and a negligible gravity effect.

Conservation of Mass Equation:

(1) $$\frac{𝜕(uA_{x})}{𝜕x}+\frac{𝜕(vA_{y})}{𝜕y}+\frac{𝜕(wA_{z})}{𝜕z}=0$$

Momentum Equations:

(2) $$uA_{x}\frac{𝜕u}{𝜕x}+vA_{y}\frac{𝜕u}{𝜕y}+wA_{z}\frac{𝜕u}{𝜕z}=-\frac{1}{\rho }\frac{𝜕p}{𝜕x}$$

(3) $$uA_{x}\frac{𝜕v}{𝜕x}+vA_{y}\frac{𝜕v}{𝜕y}+wA_{z}\frac{𝜕v}{𝜕z}=-\frac{1}{\rho }\frac{𝜕p}{𝜕y}$$

(4) $$uA_{x}\frac{𝜕w}{𝜕x}+vA_{y}\frac{𝜕w}{𝜕y}+wA_{z}\frac{𝜕w}{𝜕z}=-\frac{1}{\rho }\frac{𝜕p}{𝜕z}$$

### 2.2. Turbulence Modeling

The flow turbulence is created by using the k-omega Shear Stress Transport (SST) model. The SST model combines the advantages of k-omega and k-epsilon models, such as resolving near-wall flows and free-stream turbulence, respectively. Therefore, during simulation, key flow characteristics such as boundary layer separation, high-strain turbulence effects, and adverse pressure gradients can be predicted accurately, which contributes to the investigation of erosion in subsea valves. The SST model is based on two transport equations, the turbulence kinetic energy $\left(k\right)$ and the specific dissipation rate $\left(\omega \right)$. For steady-state conditions, as in our simulation, the equations are given as follows [32]:

Turbulence Kinetic Energy $\left(k\right)$ Equation:

(5) $$\nabla \cdot \left(\rho kv\right)=P_{k}-\beta^{*}\rho k\omega +\nabla \cdot \left[\left(\mu +\sigma_{k}\mu_{t}\right)\nabla k\right]$$

Specific Dissipation Rate (ω) Equation:

(6) $$\nabla \cdot \left(\rho \omega v\right)=\frac{\gamma }{v_{t}}P_{k}-\beta \rho \omega^{2}+\nabla \cdot \left[\left(\mu +\sigma_{w}\mu_{t}\right)\nabla \omega \right]+2\left(1-F_{1}\right)\rho \sigma_{d}\frac{1}{\omega }\nabla k\cdot \nabla \omega$$

For the near-wall, the SST model uses Low-Reynolds corrections, and in simulation, a correlation-based wall treatment is enabled, which optimizes the computing efficiency without sacrificing accuracy near the wall surface [33]. High-energy regions increase the chaotic movement of the particles, which subsequently affects the erosion severity. Therefore, the distribution of turbulent kinetic energy (TKE) is one of the outputs of the simulation, which helps to identify the critical erosion-prone areas. To avoid over-prediction of the TKE in high-strain areas, the production limiter option is activated [34].

### 2.3. Simulation Input

**Figure 1:**
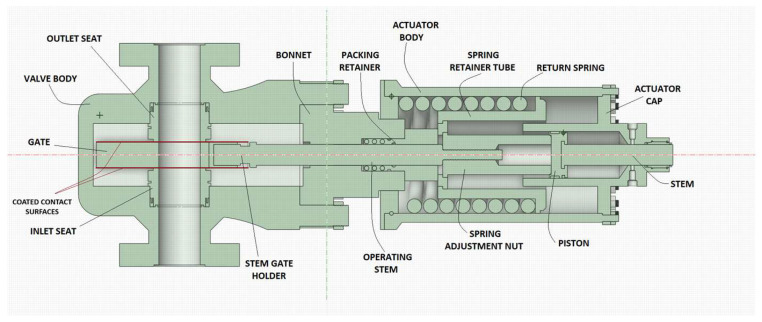
Schematic view of a subsea gate valve [35].

Subsea gate valve model and parameters. Figure 1 illustrates the model of a 5-inch subsea gate valve, which consists of two main parts: the valve itself and the hydraulic actuator. In an open position, the connection is established between the inlet and the outlet through the gate port. The pressure drop is minimal due to the straight flow path. With the closing command, the actuator pulls the gate via the stem, and the differential pressure across the valve creates a sealing force between the gate and the seats. Considering the significantly high loads and abrasive environment, the valve internals, particularly the contact surfaces of the gate and seats, are coated with hard material. The substrate material is super duplex stainless steel. Regarding the isolation from the external environment, the valve body and bonnet are bolted tight. With the packing retainer around the operating stem, the high-pressure fluid is inside the valve part.

Regarding the actuator part, the axial force required to move the gate is achieved through the piston–cylinder coupling. Hydraulic fluid from topside HPU enters the cylinder through the fluid inlet, generating force on the piston. A return spring, located between the piston retainer tube and the actuator body’s bonnet end, is compressed as the hydraulic fluid drives the piston to open the valve. The valve is kept open with the consistency of hydraulic pressure. In emergencies such as power failures, the return spring activates, moving the stem into a fail-safe position and closing the valve.

Table 1 gives details about the size, dimensions, and materials of the model used in the simulation.

Slurry test and flow parameters. API 6A standard categorizes the subsea valves into two classes based on the performance requirement [36]. The valves in class I are not intended to be used in the wells that expect high sand production, as opposed to class II valves. Therefore, the test conditions to validate the subsea valves vary depending on their class, which is a higher requirement for class II. In the research, the PDC-coated valve is intended for use in upper limit conditions; therefore, the valve is assumed to be class II. Considering that, the steps for the slurry test can be described and adjusted to apply to the simulation.

The slurry test consists of two steps. In the first step, the valve is kept fully open while sand slurry with a minimum flow of $0.30\;m^{3}/min$ circulates across the valve. The primary continuous phase of the flow is freshwater, and the sand content is adjusted to $\left(2\pm 0.5\right)\%$ and the viscosity to $\left(100_{-10}^{+20}\right)\;Pa\cdot s$, as per the ISO 10414-1 standard [37]. The size of sand particles ranges as 40–60  US mesh $\left(250–400\;\mu m\right)$. After pumping the flow for 25 h through the fully open subsea valve, the sand content and flow viscosity are measured and adjusted to the initial values again. Following that, the flow is circulated for another 25 h. After the first step, the valve is checked for leakage using fresh water and nitrogen. The second step of the slurry test uses the same properties of sand slurry in terms of the sand content and viscosity. The only difference is that the valve is cycled from fully open to a closed position at a minimum of 7 cycles/minute. When the valve is closed, the pump must create 400 psi differential pressure between valve ports. The process continues until $\left(500_{0}^{+10}\right)$ valve cycles are reached. After this, the valve is checked for leakage in a similar way as in the first step.

For the simulation, the flow-control steps can be dismissed since the software offers precise control over the parameters. This also applies to the value deviations for the values. Regarding the simulation, the objective for the first step is to estimate the erosion of the subsea valve in a fully open position after 50 h. Doing so with the short interval of reporting time $\left(1\;min\right)$ would need significant power and time. Therefore, it is decided to simulate the valve for one hour and extrapolate the results for 50 h. The same approach is applied to the second step, and the subsea valve is exposed to the sand slurry in 6 different static positions to simulate the rate of 7 cycles/minute to reach 500 cycles. Regarding the flow parameters, based on the 5-inch valve cross-section $\left(A=12.67\;mm^{2}\right)$, the inlet velocity is determined to be 0.394 m/s, and assuming the solid particles as silica sand $(density=2600\;kg/m^{3}),$ the particle mass flow rate is found to be 0.26 kg/s. Table 2 summarizes the details mentioned above.

### 2.4. Mass Loss Calculations

In the simulation model, the flow is assumed to be single-phase. Solid particles are introduced to the flow using Discrete Phase Model (DPM), which is based on the particle impact approach. Using the DPM instead of the multi-phase model results in computing power efficiency. Regarding the erosion modeling, the Oka model is integrated to evaluate the material wear under particle impacts. Equation (7) describes the Oka erosion model [38,39].

(7) $$\dot{E}=C\;\cdot \;d_{p}^{m}\cdot v_{p}^{n}\cdot f(\theta )$$

During the first step of the slurry test, the valve is kept fully open for flowing fluid, and the mass loss due to erosion during this process is determined as in Equation (8).

(8) $$∆M_{step1}={\dot{E}}_{100\%}\cdot A_{seat}\cdot t_{step1}$$

During the second step of the slurry test, the model is simulated for the dynamic valve cycles. For each position, the area-weighted erosion rates are computed using the model. Assuming the constant gate speed before/after the crack-open case, for one cycle, erosion is determined using Equation (9).

(9) $$∆M_{cycle}=\int_{0}^{8.57}\dot{E}dt\cdot \;A_{seat}$$

The mass loss during the second step of the slurry test is simply calculated by multiplying the number of cycles as described in Equation (10).

(10) $$∆M_{step2}=∆M_{cycle}\cdot N_{cycles}$$

### 2.5. Model Validity and Deviation Analysis

To verify the consistency and reliability of the simulation approach, three cumulative-exposure analyses are performed for 1 h, 10 h, and 50 h using the same steady-state flow field. These intervals represent short-, mid-, and long-term exposure durations and are selected to examine the time-dependence of erosion within the coating layer.

Both steps of the sand slurry test are simulated three times using independent random particle injection seeds for both WC and PDC materials to evaluate numerical repeatability. The relative standard deviation (RSD) is calculated according to Equation (11):

(11) $$RSD\left(\%\right)=\frac{SD}{\bar{\dot{E}}}\;\cdot \;100\%$$

As the RSD values remain below 4% for all cases, the results presented in the main manuscript are based on one representative simulation for each condition.

## 3. Results and Discussion

### 3.1. Velocity Distribution

**Figure 2:**
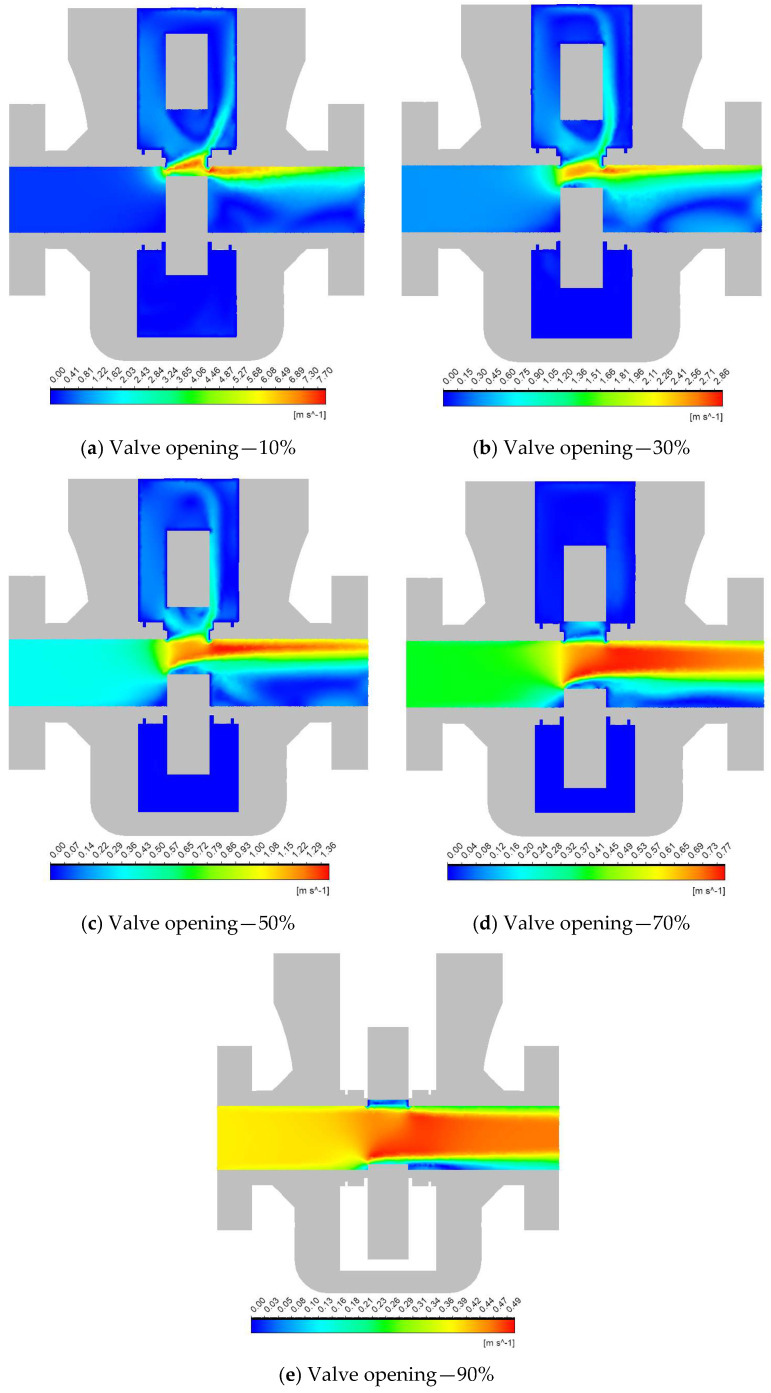
Velocity contours at different valve openings. The color scale is not uniform across.

Figure 2 shows the velocity contours illustrating the flow distribution through the subsea gate valve at different openings. Comparing different valve positions confirms the expected decrease in flow velocity as the valve opens. Additionally, one can observe the high-velocity regions, recirculating zones, and flow impingement, which are contributing factors to the erosion severity and location [40,41].

Starting with the narrower opening position, the flow reaches its maximum velocity of ≈7.8 m/s where it is highly restricted, resulting in a jetting effect, shown in Figure 2a. Due to internal wall resistivity and flow friction, the velocity drops through the internal wall. As the opening of the valve increases to 30%, the velocity gradients begin to spread over a larger area, leading to a reduced flow velocity. Near the outlet valve seat, localized high-velocity (≈2.89 m/s) can be observed as shown in Figure 2b.

Figure 2c shows the half-opening case, where the flow is more evenly distributed, reducing the intensity of velocity gradients. The peak velocity observed near the outlet seat amounts to ≈1.39 m/s. In a 70% opening, the flow exhibits a near-linear path with minimal jetting or recirculation zones. The average velocity remains moderate across the outlet port while the peak velocity value reaches ≈0.79 m/s (Figure 2d).

At a nearly fully open position (90%), as illustrated in Figure 2e, the flow is minimally restricted, which results in a uniform velocity profile across the valve. The velocity magnitude is the lowest ≈ 0.49 m/s among all cases.

### 3.2. Turbulence Analysis

**Figure 3:**
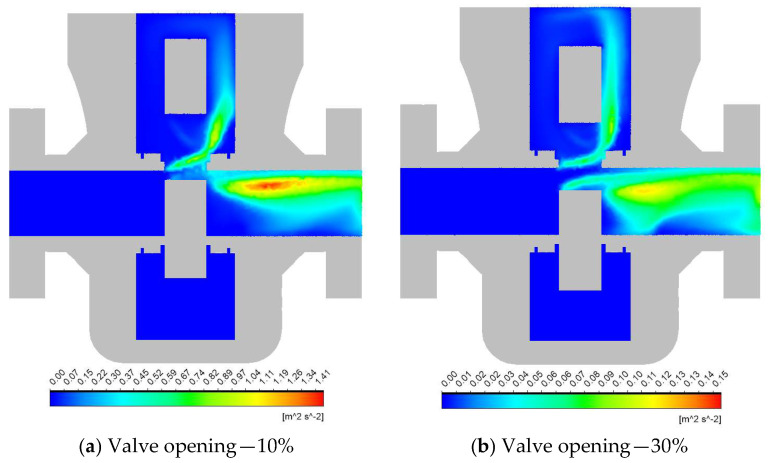

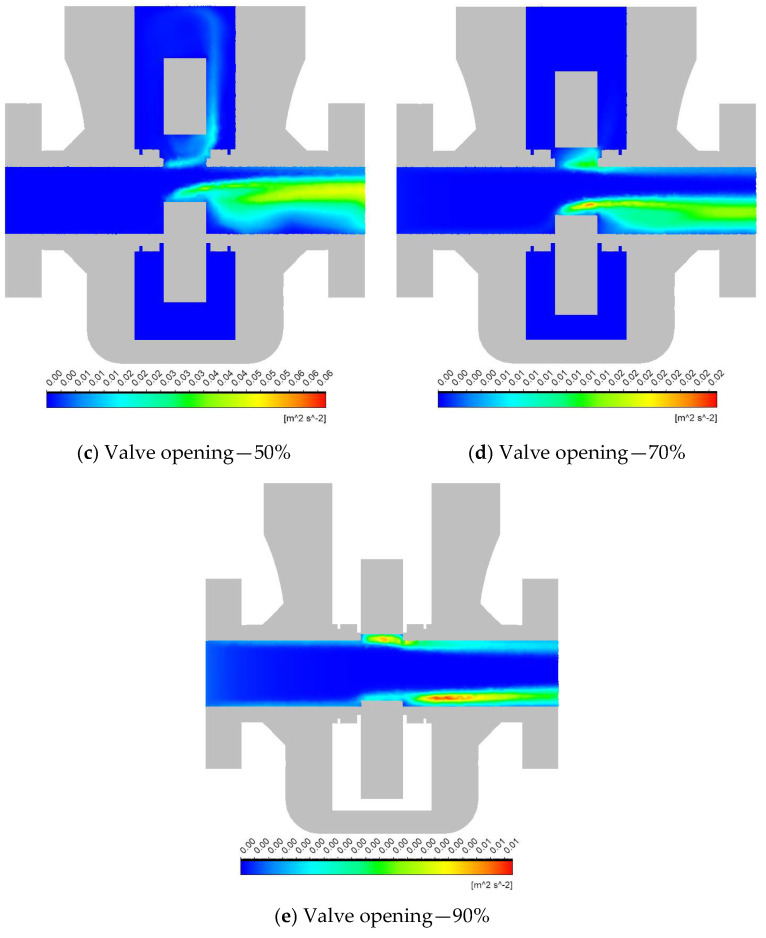
Distribution of turbulence kinetic energy at different valve openings.

The change in the distribution of TKE during valve operation is shown in Figure 3. Comparing the TKE contours with the velocity figures, it is evident that narrow openings increase the molecular viscosity of the flow, and therefore, high TKE values are not observed where the flow velocity is maximum. Instead, intense TKE $\left(\text{≈}1.44\;m^{2}/s^{2}\right)$ is concentrated in the downstream side of the gate. Additionally, one can notice that the TKE values decline faster due to wall resistance [42]. The rest of the flow area remains comparably stagnant with minimal turbulence generation (Figure 3a).

Increasing the valve opening to 30% lowers the peak TKE significantly $\left(\text{≈}0.15\;m^{2}/s^{2}\right)$. However, a moderately sized zone of elevated turbulence still appears downstream as the jet expands and interacts more broadly with the slower surrounding fluid (Figure 3b).

For the half-open case, the maximum value of TKE reduces further to $\text{≈}0.06\;m^{2}/s^{2}$ due to the larger flow area and less severe velocity changes. However, a noticeable region of elevated turbulence remains downstream of the gate, where the accelerated fluid meets slower pockets (Figure 3c).

At a 70% opening, turbulence spreads further downstream and becomes milder overall. However, the lower port of the gate acts as a restriction, and thereby creates a small turbulence zone with a peak TKE of $\text{≈}0.02\;m^{2}/s^{2}$ (Figure 3d).

When the valve is nearly fully open at 90%, the TKE across the valve is minimal. Only small patches of slightly higher turbulence $\;(\text{≈}0.01\;m^{2}/s^{2})$ are observed at the upper port of the gate and along the inner downstream wall (Figure 3e).

### 3.3. Erosion Rate Distribution

**Figure 4:**
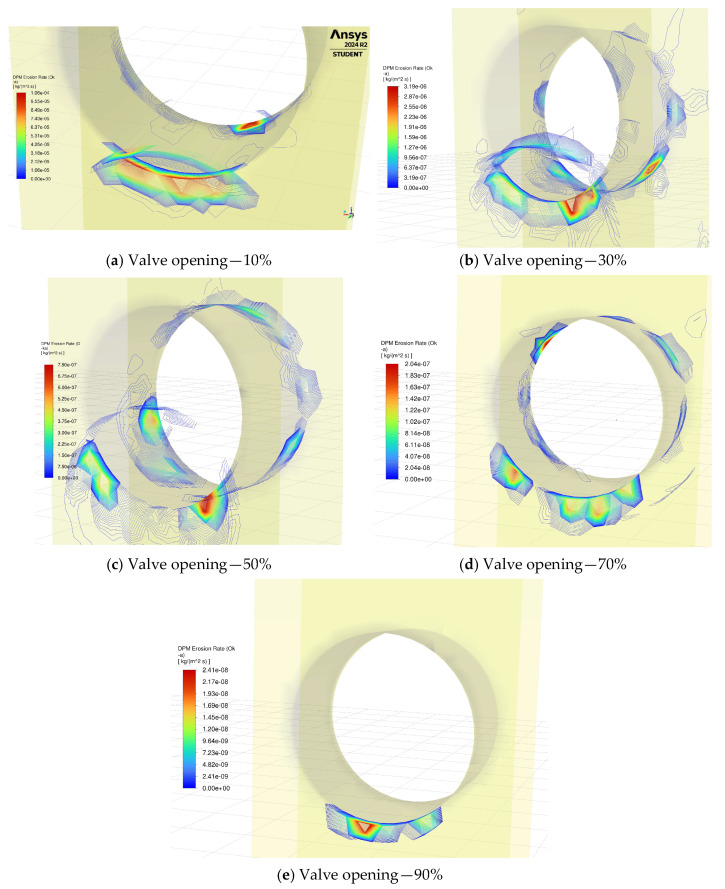
Erosion rate contours at different valve openings.

Regarding the erosion of the coating material in the valve, Figure 4 highlights the erosion rate at the different openings during valve operation. The most severe and localized erosion happens at 10% opening, where the high-velocity jet causes intense particle impacts on the valve seats. Due to the intense turbulence on the downstream side, the outlet gate seats are eroded at a similar rate. Figure 4a shows the maximum erosion rate equal to $106\;mg/m^{2}\cdot s$. As the valve opens to 30%, the erosion begins to spread slightly along the valve seat and nearby areas due to the increased flow passage, although the peak rate drops significantly to $\;3.19\;mg/m^{2}\cdot s$ as shown in Figure 4b. In the half-open case, the erosion distribution becomes broader with the impact region spread inside the gate port and gate seats. Figure 4c highlights the maximum erosion happening at the seat with the rate of 0.75$\;mg/m^{2}\cdot s$. The erosion intensity decreases compared to smaller openings as the particle velocities are reduced. With a 70% opening, the erosion rate peak decreases further to $0.2\;mg/m^{2}\cdot s$ (Figure 4d). At 90%, the valve experiences the most uniform and minimal erosion distribution, as the nearly unrestricted flow reduces velocity gradients and diminishes particle impacts on the surfaces. As a result, a maximum $0.02\;mg/m^{2}\cdot s$ erosion rate is recorded in Figure 4e. The results show a clear trend of erosion transitioning from highly localized and severe at smaller openings to more distributed and less intense at larger openings. The same procedure is repeated for the PDC-coated valve, and the results of maximum erosion rates during valve operation are summarized in Table 3 for both cases. The results indicate that replacing the WC coating material with PDC material leads to an erosion rate reduction of 64% and 5% at the minimum and maximum opening cases, respectively.

### 3.4. Cumulative Erosion and Mass Loss

**Figure 5:**
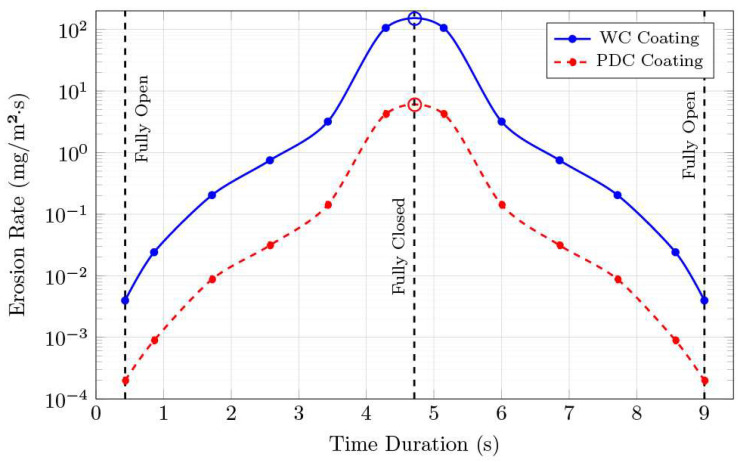
Erosion rate vs. time duration for one valve cycle (logarithmic *y*-axis). Fully open and fully closed positions are highlighted.

Section 3.3 determines the erosion rates at six static positions for the subsea gate valve. Figure 5 plots the results versus valve opening with interpolation and compares the WC and PDC coating valves. As can be seen, the erosion rate significantly increases as the valve near “crack-open” and “crack-close” moments.

Following the steps covered in Section 2.4, the results from the simulation are used in Equation (8). The right side of the equation is solved using Simpson’s rule as giving cumulative erosion values for both materials (Table 4):

$$\int_{0}^{8.57}{\dot{E}}_{wc}\left(t\right)\;dt\;\text{≈}\;188.73\;mg/m^{2}$$

$$\int_{0}^{8.57}{\dot{E}}_{pdc}\left(t\right)\;dt\;\text{≈}\;7.58\;mg/m^{2}$$

By having the cumulative erosion values, the mass loss during each step of the slurry test is determined as in Equations (9) and (10), and the results are summarized in Table 5.

### 3.5. Time-Dependence and Repeatability Analysis

To assess the influence of the exposure duration on erosion progression, additional cumulative-exposure analyses are performed for 1 h, 10 h, and 50 h using the same steady-state flow field as in the base simulation. These analyses provide short-term, mid-term, and long-term representations of the coating response under identical boundary conditions.

The results showed that the Step 1 erosion varied by less than 3% for both WC and PDC coatings across these intervals, confirming consistent wear behavior over time. When compared with the total erosion including Step 2, the variation was below 0.05%, demonstrating that the effect of exposure duration is insignificant in the overall erosion performance. This validates the use of short-term data for long-term extrapolation in our case. Detailed results are presented in Table A2 (Appendix A).

Each step of the sand slurry test is repeated three times for both materials using independent random particle-injection seeds to quantify numerical variability. The RSD of the maximum erosion rate is below 4% for both materials. These small deviations confirm that the solver predictions are numerically stable and reproducible. Table A3 lists the mean ± SD values of cumulative erosion for each case.

### 3.6. Coating Failure Mechanism and Erosion Trend

The erosion trends reported here align with established failure modes of the two coatings. For WC, impact-induced stresses foster microcrack initiation at carbide–binder interfaces with subsequent grain pull-out, promoting roughening and higher local removal where particles impinge near the seat edges. For PDC, the continuous diamond network, higher hardness, and lower friction reduce abrasive retention and suppress crack growth, so material removal is largely limited to localized spallation events. These mechanisms, also observed in our prior microscopy [13] and reported in the literature for PDC and cemented carbides, are consistent with the lower erosion magnitudes and extended lifetime predicted for the PDC-coated valve.

### 3.7. Expected Lifetime of a Subsea Valve

In the same conditions, the results show that we can expect 5.407 mg and 715.7 mg mass loss of WC material after the first and second steps of the slurry test, which makes the total mass loss 721.1 mg. For the PDC material, the numbers are significantly less, equal to 0.273 mg and 28.74 mg after each step of the slurry test. The PDC coating lost 29.01 mg of its mass during the whole test. However, due to density differences, mass loss is not the best way of comparing the performance of both materials. The eroded depth is determined using Equation (12).

(12) $$h_{loss}=\frac{∆M_{sum}}{Density_{material}\cdot A_{seat}}$$

$$h_{loss},wc=6.095\;\mu m$$

$$h_{loss,\;PDC}=1.093\;\mu m$$

Thereby, during the slurry test, 6.095 μm of the WC coating and 1.093 μm of the PDC coating material were lost. This means that at the end of the slurry test, the PDC material indicates a 5.6 times less eroded depth than the WC material. Applying the results in practice, the subsea valves are designed to withstand for 25 years. Assuming equal valve cycles annually during 25 years, the yearly loss depth is determined as per Equation (13), where the results are 8.249 μm for WC and 1.845 μm for PDC material.

(13) $${\dot{h}}_{loss}=\frac{1}{\rho_{m}\cdot A_{seat}}\;\left(∆M_{step1}\cdot \frac{1\;year}{50\;h}+∆M_{step2}\cdot \frac{1\;year}{25\;years}\right)$$

$${\dot{h}}_{loss,\;WC}=8.249\;\mu m/year$$

$${\dot{h}}_{loss,PDC}=1.845\;\mu m/year$$

The API 6A standard recommends a minimum coating thickness of 25 μm for subsea valves [36]. Considering the same exposed conditions, the minimum thickness will be fully eroded in around 3 years for the WC coating and around 13.5 years for the PDC coating.

From a practical point of view, the conditions that the valve is expected to withstand during its lifetime are significantly milder than the ones we are exposed to during the slurry test. However, the results may be a new reality for future applications in fields with high requirements. Comparing the two materials, it is evident that PDC material outperformed WC coating material by slowing the erosion rate of the coating thickness by approximately 77.6%. As a result, the subsea valve with PDC coating offers 4.5 times greater lifetime than the WC material with the same coating thickness.

The overall results reconfirm our expectations about the performance of the PDC material in subsea valves and help us visualize how the erosion rate changes depending on the gate position, flow velocity, and turbulent kinetic energy. Additionally, the simulation shows us the areas where intense erosion is happening and the corresponding eroded depth, which is used in determining the expected lifetime of the subsea valve.

### 3.8. Limitations

- The erosion predictions for 50 h are extrapolated from a one-hour simulation. Although this approach initially assumes a near-linear wear progression, additional cumulative-exposure analyses at 1 h, 10 h, and 50 h confirm that the variation in erosion remained within 3%. This validates the use of short-term results for long-term estimation.
- To assess numerical repeatability, each step of the slurry test is repeated three times using independent random particle-injection seeds. The relative standard deviation (RSD) remains below 4% for all cases, which confirms stable and reproducible solver performance.
- The Oka erosion model was selected due to its established performance across ductile and brittle materials, including its consideration of impact angles. However, like all empirical models, it has limitations under complex geometries and variable flow conditions.
- The simulation uses the DPM with particle sizes ranging as 250–400 μm. Although a size range is considered, broader variations in subsea environments are not fully represented.
- Full dynamic simulation of 500 valve cycles was not feasible. Instead, six representative static positions were selected, including critical stages such as crack-open and crack-close. Interpolation (Simpson’s rule) was applied to estimate cumulative erosion. While this introduces approximation, the method is conservative and consistent with practices in valve erosion studies.
- The current study is part of a pre-prototype design phase. While experimental validation is not included in this study, a physical test is planned further. The numerical results will be validated in future work using gravimetric mass-loss measurements under API 6A slurry test conditions. The test setup will follow the same flow geometry and particle loading as in the simulations. A precision balance (±0.01 mg) will be used to determine cumulative material loss after each test.
- The simulations are based on API 6A Class II slurry test conditions. Broader operating conditions such as different flow rates, particle distributions, and slurry compositions, are not addressed in this work.
- Manufacturing PDC-coated valve components can be challenging due to the HPHT process and residual stresses, but these factors are well managed with current industrial methods and do not pose a major limitation.
- Post-erosion SEM/TEM of valve components is not performed in this simulation stage and is planned in the prototype validation to directly verify the coating damage modes.

## 4. Conclusions

- This study presents a quantitative comparison of WC and PDC coatings under API slurry test conditions, which enables the prediction of erosion behavior and valve lifetime in realistic subsea environments.
- Severe erosion occurs at narrow valve openings (10–30%). At these openings, the flow velocity reaches up to 7.8 m/s and the turbulence kinetic energy peaks at 1.44 m^2^/s^2^, resulting in highly localized erosion, especially on the valve seats.
- Jetting effects at partial openings cause aggressive surface wear. Measured erosion rates are 106 mg/m^2^ s for tungsten carbide (WC) coatings and 4.24 mg/m^2^ s for polycrystalline diamond compact (PDC) coatings.
- At near-full opening (90%), flow velocity drops to 0.49 m/s and turbulence kinetic energy decreases to 0.01 m^2^/s^2^, resulting in a significantly lower erosion risk.
- PDC coatings demonstrate superior erosion resistance. Simulation-based extrapolation shows that only 1.093 μm of the PDC layer is removed after extended slurry exposure—5.6 times less than the erosion observed in WC coated surfaces.
- Applying erosion trends to coating thickness standards (based on American Petroleum Institute guidelines), PDC coatings reduce the total erosion depth by 77.6% and increase the valve’s expected lifetime by a factor of 4.5.
- Repeatability analyses confirmed numerical stability, with relative standard deviations below 4%, reinforcing the robustness of the simulation framework.
- The findings elevate the technology readiness level (TRL) of PDC-coated subsea valves, supporting their application in offshore field developments with improved operational reliability.
- Improved erosion resistance and extended service life of PDC-coated valves contribute to reduced offshore project costs, lower intervention frequency, and decreased health, safety, and environmental (HSE) risks.

## Figures and Tables

**Table 1:** Parameters of the subsea gate valve model.

Parameter	Value	Parameter	Value
Face-to-face dimension	686.1 mm	Gate seat area	$7583.7\;mm^{2}$
Body width	920 mm	Substrate material	25Cr super duplex
Bore diameter	127.8 mm	Material density (WC)	$15,600\;kg/m^{3}$
Gate port diameter	128.6 mm	Material density (PDC)	$3500\;kg/m^{3}$

**Table 2:** Simulation flow setup and parameters.

Parameter	Value	Parameter	Value
Fluid phase	Freshwater	Particle phase	Silica sand
Fluid density	$1000\;kg/m^{3}$	Particle density	$2600\;kg/m^{3}$
Fluid viscosity	0.001 kg/m·s	Particle diameter	250–400 μm
Flow rate	$0.30\;m^{3}/min$	Particle mass flow rate	0.26 kg/s
Inlet velocity	0.394 m/s	Pressure (closed)	400 psi

**Table 3:** Maximum erosion rates for WC and PDC coatings at different valve openings.

Opening (%)	Max. Erosion Rate WC Coating (mg/m^2^ ∙ s)	Max. Erosion Rate WC Coating (mg/m^2^ ∙ s)
10	$1.06\times 10^{2}$	$4.24\times 10^{0}$
30	$3.19\times 10^{0}$	$\;1.42\times 10^{-1}$
50	$\;7.50\times 10^{-1}$	$\;3.12\times 10^{-2}$
70	$\;2.04\times 10^{-1}$	$\;8.74\times 10^{-3}$
90	$\;2.41\times 10^{-2}$	$\;8.91\times 10^{-4}$
100	$\;3.96\times 10^{-3}$	1.98$\;\times 10^{-4}$

**Table 4:** Cumulative erosion of WC and PDC material after each step of the slurry test.

Step No.	Flowrate $\left(m^{3}/min\right)$	Gate Position	Duration (Hours)	$E_{wc}$ g/m^2^	$E_{PDC}$ g/m^2^
1	0.300	fully open	50.00	0.713	0.036
2	0.300	7 cycles/min	1.190	94.37	3.790

**Table 5:** Mass loss of WC and PDC material after each step of the slurry test.

	A Cycle	Slurry Test Step 1	Slurry Test Step 2	Slurry Test SUM
$∆M_{wc}(mg)$	1.431	5.407	715.7	721.1
$∆M_{PDC}(mg)$	0.057	0.273	28.74	29.01

## Data Availability

The original contributions presented in this study are included in the article. Further inquiries can be directed to the corresponding author.

## Nomenclature and Acronyms

Nomenclature	
$A_{seat}$	Area of valve seat
$A_{x},A_{y},A_{z}$	Area fractions in x, y, z directions
C	Material constant
$d_{p}$	Particle diameter
$F_{1}$	Blending function
$h_{loss}$	Depth of erosion
${\dot{h}}_{loss}$	Depth of erosion per year
k, TKE	Turbulent kinetic energy
m	Particle size exponent
$N_{cycles}$	Number of valve cycles
p	Pressure
u,v,w	Velocity in x, y, z directions
$v_{p}$	Particle velocity
$v_{t}$	Turbulent kinetic viscosity
$\dot{E}$	Erosion rate
∆M	Mass loss due to erosion
β	Model constant
γ	Model constant
f(θ)	Impact angle function
μ	Dynamic viscosity
$\mu_{t}$	Turbulent viscosity
ρ	Fluid density
$\rho_{m}$	Material density
$\sigma_{k,w,d}$	Model constants
w	Specific dissipation rate
Acronyms	
API	Area of valve seat
CFD	Area fractions in x, y, z directions
DPM	Material constant
HPHT	Particle diameter
HSE	Blending function
HVOF	High-Velocity Oxy-Fuel (HVOF)
PCD	Polycrystalline diamond
PDC	Depth of erosion
RANS	Depth of erosion per year
RSD	Relative standard deviation
SST	Turbulent kinetic energy
SD	Standard deviation
TRL	Particle size exponent
WC	Number of valve cycles

## Appendix A

**Table A1:** Fundamental mechanical and thermal properties of cemented WC and PDC coatings relevant to subsea valve applications.

Property	WC	PDC	Typical Test/Standard	Reference
Vickers hardness (HV)	1200–1600	7000–9000	ASTM E384 [43]	[15,22,23]
Rockwell hardness (HRC, eq.)	85–92	>100 (est.)	ASTM E18 [44]	[14,22]
Coefficient of friction (self-paired)	0.12–0.20	0.05–0.08	ASTM G99 [45]	[13,23]
Thermal conductivity (W m^−1^ K^−1^)	84–110	600–1200	ASTM E1461 [46]/ISO 22007-2 [47]	[16,22]
Thermal expansion coefficient (×10^−6^ K^−1^)	4.3–6.0	1.0–1.2	ASTM E228 [48]	[17,24]
Typical manufacturing process	HVOF/Plasma-sprayed WC–Co	HPHT sintered diamond on WC substrate	-	[19,20,21]
Adhesion strength (MPa)	60–80 (HVOF)	>80 (HPHT bonded)	ASTM C633 [49]/Scratch test ISO 20502 [50]	[18,24]
Thermal stability limit (°C)	≤500–600 (binder softening)	700–1200 (depends on binder)	-	[22,25]

**Table A2:** Step 1 erosion mass loss of WC and PDC materials for 1 h-, 10 h-, and 50 h-based simulations (50 h-duration). The hyphen (-) denotes the baseline reference used for calculating the percentage variation.

	Step 1 (1 h-Based)/(Variation %)	Step 1 (10 h-Based)/(Variation %)	Step 1 (50 h-Based)/(Variation %)
$∆M_{wc}(mg)$	5.407 (-)	5.489 (+1.52%)	5.533 (+2.33%)
$∆M_{PDC}(mg)$	0.273 (-)	0.278 (+1.83%)	0.281 (+2.93%)

**Table A3:** Mean ± SD cumulative erosion and relative standard deviation (RSD) for repeated simulations.

Material	Step	Mean Erosion Mass Loss (mg)	SD (mg)	RSD (%)	Number of Repetitions
WC	Step 1 (fully open)	5.428	0.079	1.45	3
	Step 2 (cycling)	720.4	21.8	3.03	3
PDC	Step 1 (fully open)	0.275	0.009	3.18	3
	Step 2 (cycling)	28.763	0.846	2.94	3
